# Hashimoto’s Thyroiditis and the Risk of Papillary Thyroid Cancer in Children

**DOI:** 10.3390/cancers15194902

**Published:** 2023-10-09

**Authors:** Jean-Nicolas Gallant, Vivian L. Weiss, Sheau-Chiann Chen, Jiancong Liang, Ryan H. Belcher, Fei Ye, Hernan Correa, Huiying Wang

**Affiliations:** 1 Department of Otolaryngology—Head and Neck Surgery, Vanderbilt University Medical Center, Nashville, TN 37232, USA; 2 Department of Pathology, Microbiology and Immunology, Vanderbilt University Medical Center, Nashville, TN 37232, USA; 3 Department of Biostatistics, Vanderbilt University Medical Center, Nashville, TN 37232, USA; 4 Monroe Carell Jr. Children’s Hospital, Vanderbilt University Medical Center, Nashville, TN 37232, USA

**Keywords:** Hashimoto’s thyroiditis, pediatric, children, thyroid nodule, papillary thyroid carcinoma, thyroid cancer

## Abstract

**Simple Summary:**

Hashimoto’s thyroiditis (HT) is an autoimmune disease of the thyroid that has been associated with the development of thyroid cancer in certain adult populations. This study sought to determine whether there was a similar link between pediatric thyroid cancer and HT. Data from this study suggest that children with thyroid cancer are more likely to have HT—although this does not seem to affect their outcomes or survival. These findings are important as they may help risk-stratify children who present with a thyroid nodule.

**Abstract:**

The association between Hashimoto’s thyroiditis (HT) and pediatric thyroid cancer is controversial. Most studies examining this connection have been based on adults, and larger studies in children are lacking. We performed a retrospective study of all sequential pediatric patients who underwent a thyroidectomy for a neoplasm at our institution over a twenty-year period in order to explore the link between HT and pediatric thyroid cancer. A total of 153 patients, median age 16.5 (interquartile range [IQR] 14.2–18.3) years, underwent thyroid surgery for a neoplasm. Patients were mainly female (80%) and White (84%). Median follow-up was 58.6 (IQR 20.7–105.4) months. Thirty-five (23%) patients had HT. Patients who underwent thyroid surgery and had HT were more likely to harbor a malignant neoplasm (*p* = 0.05); specifically, papillary thyroid carcinoma (PTC, *p* = 0.02). There was a difference in the distribution of HT among the subtypes of PTC (*p* = 0.03). Despite this, there was no difference in terms of survival between patients with/without HT. In conclusion, children with a thyroid malignancy, specifically, PTC, are more likely to have HT. The association between HT and pediatric PTC appears to be subtype-specific but does not seem to affect patient survival.

## 1. Introduction

The worldwide incidence of pediatric thyroid cancer continues to rise, and it is currently the eighth most frequently diagnosed (incident) malignancy in children and the second most common (prevalent) cancer among adolescent girls [1,2,3,4]. Pediatric thyroid cancers typically present as thyroid nodules, which are less common in children than in adults (~2% of children versus ~30% of adults) [5,6]; however, when present, they carry a greater risk of malignancy compared to those in adults (~25% of nodules are malignant in children versus ~5% in adults) [7,8].

Due to the increased probability of malignancy in children, current recommendations are to proceed with diagnostic lobectomy for nodules of indeterminate malignant potential—which accounts for about 20% of pediatric thyroid nodules [9]. Lobectomy, the removal of the thyroid lobe containing the nodule in question, allows for histologic assessment (surgical pathology), the reference standard for the final diagnosis of a thyroid nodule. Unfortunately, thyroid surgery in children has a significantly higher rate of complications than in adults [10,11].

The majority (60–90%) of pediatric thyroid cancers are papillary thyroid cancers (PTCs); follicular (FTC) and medullary thyroid cancers (MTC) make up 5–10% of pediatric thyroid cancers [9]. Correspondingly, the majority of pediatric thyroidectomies are performed for differentiated thyroid cancer (~40%; either PTC or FTC), a thyroid nodule (~25%; which can be benign), Grave’s diseases (~20%), multinodular goiter (~15%), or prophylactically for patients with multiple endocrine neoplasia syndrome (~5%; which are associated with MTC) [12].

Given the mounting incidence and prevalence of pediatric thyroid cancer and the increased risks of pediatric—as opposed to adult—thyroid surgery, there is a need for better predictors of malignancy. While there have been successful efforts at applying adult imaging, cytologic, and genetic tests for use in children [13], there are few clinical risk factors associated with pediatric thyroid cancer. Outside of hereditary cancers (such as MTC associated with multiple endocrine neoplasia syndrome), the majority of pediatric thyroid cancers are sporadic in nature. The only consistently identified risk factor is childhood radiation exposure—presumably because thyroid tissue is more radiation-sensitive at a younger age [14]. As such, while it is known that there is about a 10-fold increase in the risk of thyroid cancer in childhood cancer survivors treated with radiation [15], there are no other validated clinical risk factors that have been identified for pediatric thyroid cancer.

Given that chronic inflammation is a hallmark of cancer [16,17,18], it has long been proposed that thyroid cancer could be caused by Hashimoto’s thyroiditis (HT) [19], also known as chronic autoimmune thyroiditis. While this hypothesis has been supported by large (tens of thousands of patients) meta-analyses in adults [20,21], the association between HT and thyroid cancer remains controversial. In children, the link between HT and thyroid cancer is even more tenuous given the smaller number of patients (hundreds) across several studies [22,23,24].

The goal of this study was to investigate the association between HT and thyroid cancer in children by leveraging a large surgical patient cohort. We hypothesized that children with HT were more likely to have thyroid cancer and that this would lead to worse progression-free survival.

## 2. Materials and Methods

### 2.1. Study Design

This was an Institutional Review Board (IRB)-approved retrospective study. A waiver of informed consent was granted by the IRB owing to the retrospective nature of this study. This study adheres to the Strengthening the Reporting of Observational Studies in Epidemiology (STROBE) reporting guidelines [25].

### 2.2. Study Population

All consecutive patients who underwent surgery for a thyroid neoplasm at the Monroe Carell Junior Children’s Hospital at Vanderbilt University Medical Center between 2003 and 2022 were considered for this study if they were 21 years of age or younger at the time of surgery. All patients had thyroid nodules that were diagnosed pre-operatively using clinical examination, imaging, and/or fine needle aspiration biopsy. Patients harboring both benign (e.g., adenoma) and malignant (e.g., papillary thyroid carcinoma) neoplasms (on final surgical histopathology) were included. Patients with multinodular goiter were excluded. Three patients with PTCs were excluded: two with their cancers arising in thyroglossal duct cysts without involvement of the thyroid gland and one with a germline *TMEM127* mutation [26]. Patients with MTC and poorly differentiated thyroid carcinoma (PDTC) were excluded due to their low numbers, differing biology, and skewed patient demographics (e.g., MTC was overwhelmingly found in younger male patients).

### 2.3. Review of Papillary Thyroid Carcinoma Pathology

Given changes in diagnostic criteria over the 20-year study period, the histopathology of all patients with PTC was re-reviewed by two pediatric pathologists (JL and HW) to confirm the original diagnosis or to re-classify the specimens per current diagnostic criteria (World Health Organization Classification of Tumours, 5th Edition) [27]. 

### 2.4. Diagnosis of Hashimoto’s Thyroiditis

We defined HT as the presence of both (1) diffuse lymphocytic infiltration with germinal center formation and Hürthle cell metaplasia in surgical resection specimens and (2) a positive clinical history (with or without positive laboratory testing).

### 2.5. Additional Clinical Data

Patient demographics, clinical histories, treatments, and outcomes were manually collected from the electronic health record. Tumor pathology was classified according to the American Joint Committee on Cancer (AJCC) 8th edition [28]. American Thyroid Association (ATA) risk categories were determined using the latest pediatric guidelines and the definition of extensive disease provided in the adult guidelines (extensive involvement if >5 lymph nodes or size of ≥3 cm in largest diameter) [9,29].

For progression-free survival (PFS) analyses, the interval between the completion of initial therapy to the date of progression was calculated. The date of disease progression was determined by the first date of either an increasing thyroglubilin (Tg, in an appropriately thyroid stimulating hormone suppressed patient) or the increase in size of a lesion by imaging. All patients that were determined to be progressive by Tg if they had subsequent imaging evidence of progressive disease. For patients without progression, the date of last follow-up was used to determine PFS—and the data appropriately censored. Patients with no follow-up after therapy were omitted from survival analyses. 

Outcomes were defined roughly in line with the ATA. Specifically, ‘Good’ outcomes included patients with no evidence of disease (NED), defined by undetectable thyroglobulin (Tg) level, lack of circulating anti-thyroglobulin antibodies (anti-Tg), and a thyroid ultrasound indicating no evidence of disease. Patients without imaging follow-up were determined to be NED using laboratory testing (undetectable Tg or anti-Tg) alone. Imaging alone (without labs) was only sufficient for NED if the patient had a hemithyroidectomy or no RAI. ‘Indeterminate’ outcomes were defined by stable/detectable Tg < 1.0 ng/mL, stimulated Tg < 10 ng/mL, positive anti-Tg levels that were not increasing, imaging without Tg labs, and/or inconclusive imaging. ‘Poor’ outcomes included persistent or recurrent disease. Persistent disease included stable Tg > 1.0 ng/mL, stimulated Tg > 10 ng/mL, and/or a persistent lesion by imaging that did not increase in size over time. Recurrent disease included malignancies that could be measured (via imaging or laboratory testing) after a designation of NED but were treated with local intervention and followed by stable or decreasing tumor size or Tg.

### 2.6. Statistical Analyses

Univariate tests were performed using Welch’s two sample *t*-test for continuous variables (analyzed on a natural log scale) and the chi-square test or Fisher’s exact test categorical variables. Progression-free survival rates were estimated using the HT status with the Kaplan–Meier method. Hazard ratios are reported with 95% confidence intervals. The proportional hazards (PH) assumption was tested and graphically assessed using the scaled Schoenfeld residuals. All statistical tests were two-sided, with a significance level of 0.05; no adjustment of multiple hypothesis testing was performed. All statistical analyses were conducted using R (R version 4.1.2, The R Foundation for Statistical Computing, Vienna, Austria).

## 3. Results

### 3.1. Patient Characteristics

A total of 153 patients underwent surgery for a thyroid neoplasm (Table 1). The median age at surgery was 16.5 (interquartile range [IQR] 14.2–18.3) years. Patients were mainly female (*n* = 123, 80%) and White (*n* = 128, 84%). The median follow-up time was 58.6 (IQR 20.7–105.4) months. Thirty-five (23%) patients had evidence of chronic lymphocytic thyroiditis on surgical pathology in addition to a clinical history consistent with HT. These patients did not differ in terms of any measured demographic; however, they did have more frequent alterations in thyroid serologies (*p* < 0.001), as expected.

### 3.2. Association of HT and PTC

Patients with HT were more likely to harbor a malignancy in their nodules (*p* = 0.05, Table 2). These cancers were not significantly different in terms of size (*p* = 0.63) or nodal staging (*p* = 0.20). Those with HT were more likely to have PTC as compared to other pathologies (*p* = 0.02); however, the statistical significance of this finding was diminished when considering all thyroid neoplasms individually (*p* = 0.12). 

There was a difference in the distribution of HT among patients with PTC (*p* = 0.03, Table 3). Remarkably, follicular variant (FV) PTC (*n* = 10) was only diagnosed in patients without HT. Interestingly, the diffuse sclerosing (DS) variant of PTC (*n* = 5) was mainly diagnosed in patients with HT (5/8 patients; 63%). There was no difference when comparing classical PTC as opposed to all other subtypes (*p* = 0.98).

### 3.3. Association of HT and Patient Cancer Outcomes

**Figure 1:**
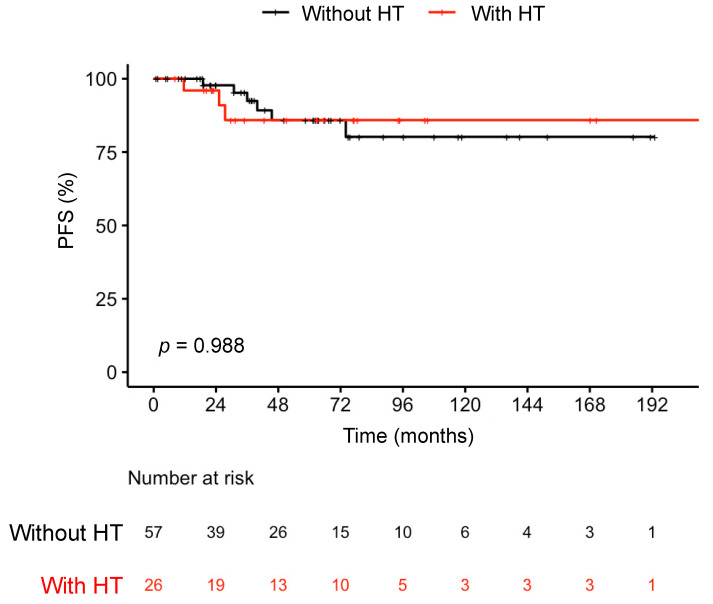
Survival of pediatric thyroid cancer patients with/out Hashimoto’s thyroiditis. Abbreviations: HT, Hashimotos’ thyroiditis; PFS, progression-free survival; *p*, *p*-value from log-rank test used to compare the PFS of groups.

Eighty-three patients with thyroid cancer, including 26 (31%) patients with HT, had outcome data (Table 4). The remaining 23 patients were lost to follow-up after a single post-operative visit. As with our main cohort, this subset of thyroid cancer patients did not differ in terms of nodule risk categories (Table S1). Patients received similar amounts of surgery, regardless of whether they had HT or not. Patients had similar outcomes (*p* = 0.08), and there was no difference in terms of median progression-free survival (hazard ratio = 1.01 [0.25–4.1], Figure 1).

### 3.4. Association of HT and PTC Patient Outcomes

Given the heterogeneity of our cancer-patient cohort, we took a closer look at patients with the most common cancer, classic PTC. Of the 59 patients with classic PTC and outcome data, 18 (31%) had a diagnosis of HT. Interestingly, HT appeared to be protective in this context: patients with classic PTC had more aggressive pathology and worse disease control than those with classic PTC and HT (Table S2). However, as with our overall cohort, there was no difference in terms of the median progression-free survival (hazard ratio = 0.65 [0.08–5.1], Figure S1)—although the sample size was smaller.

## 4. Discussion

By leveraging a large surgical cohort of pediatric thyroidectomy patients, we were able to find an association between HT and childhood thyroid cancer. That is, we found that patients with pediatric thyroid cancer, most commonly, PTC, were significantly more likely to have concurrent HT. Among those patients with PTC, we found that HT was not evenly distributed, with, for example, FVPTC only being diagnosed in patients without HT. Most importantly, despite these differences, we found that there did not appear to be a significant difference in survival in pediatric thyroid cancer patients with HT—with nearly 5 years (58.6 months) of median follow-up time. 

The association between pediatric HT and thyroid cancer has been studied for several decades [30]. Most studies have found an association between HT and the risk of pediatric thyroid cancer [24,31,32,33]. However, few studies have examined clinicopathologic characteristics and patient outcomes—such as the present study. Published data are mixed, with some suggesting that HT leads to more aggressive thyroid cancers [30,34,35], and other data suggesting that it makes no difference—or that HT may be protective [23,36,37].

The potential discrepancies between studies on HT and thyroid nodules in pediatric patients may have to do with how HT and malignancy are defined. While the clinical diagnosis of HT often is deemed one of the most straightforward in endocrinology, it is not without nuance or controversy [38]. HT can be diagnosed using a clinical history, laboratory testing, imaging, and/or pathology—with histopathology considered the diagnostic ‘gold standard’ [39,40]. In addition to this standard, we used clinical history (with or without diagnostic serologies) in order to define HT in a strict manner. However, as shown by the data in Table 1, while some patients had some features of HT, they could not be given the diagnosis of HT because of our strict definition. 

Another potential difference with prior studies and the current one has to do with how cancer is defined. In the current study, we only used histopathology, the ‘gold standard’ for the diagnosis of thyroid malignancies [26]. Other high-quality studies have used surrogates of histopathology, such as fine needle aspiration (FNA) biopsy [24]. However, without surgical pathology, we would not have been able to draw a link between HT and diffuse sclerosing and follicular-variant PTC. The association of DSPTC and HT is intriguing, given the lymphocytic infiltrate frequently seen on histopathology with DSPTC and the more aggressive nature of this malignancy [41,42]. The association between FVPTC and pediatric patients without HT is also interesting, and, to our knowledge, has never been reported. Still, these subtypes are relatively rare, and we were not able to discern a survival difference in patients with these cancers and with/out HT. While the surgical design of our study is a strength, it could also be considered a weakness: one important limitation of this study includes surgical ascertainment bias, which could inflate the association between thyroid cancer and HT [24,43]. We tried to minimize this potential bias by including all consecutive thyroidectomies for a neoplasm (whether benign or malignant) over a 20-year period.

There are several other limitations to the current study. The retrospective observational nature of the study makes it impossible to determine the causality of the association between HT and pediatric thyroid cancer. It is conceivable that chronic low-level inflammation from HT predisposes patients to pediatric thyroid cancer; it is also conceivable that pediatric thyroid cancer itself leads to inflammation and resultant lymphocytic infiltration. While all patients in our cohort with a diagnosis of HT had lymphocytic thyroiditis on histopathology, several patients without HT also had lymphocytic thyroiditis on histopathology (as discussed above). Likewise, while all patients with HT had a clinical history of hypothyroidism or abnormal serologies, so did several patients without HT. Hence, additional large prospective studies of pediatric patients with HT are needed to determine their relative risk of developing thyroid cancer and whether this warrants a change in patient management. Such prospective studies have been performed in adults [44] and are warranted in children.

## 5. Conclusions

Pediatric patients with a thyroid malignancy, specifically, PTC, are more likely to have concurrent HT. Additional prospective studies are needed to better determine this link, its causality, and how this may affect patient outcomes.

## Figures and Tables

**Table 1:** Patient characteristics.

Variable	All Patients (*n* = 153)	Without HT (*n* = 118)	With HT (*n* = 35)	Statistic
Age at surgery, years, median (Q_1_, Q_3_)	16.5 (14.2, 18.3)	16.8 (14.2, 18.6)	16.1 (13.9, 17.2)	*p* = 0.22 ^a^
Sex, *n* (%)				*p* = 0.37 ^b^
Female	123 (80.4)	93 (78.8)	30 (85.7)	
Male	30 (19.6)	25 (21.2)	5 (14.3)	
Race/ethnicity, *n* (%)				*p* = 0.40 ^c^
White	128 (83.7)	99 (83.9)	29 (82.9)	
Black	11 (7.2)	10 (8.5)	1 (2.9)	
Hispanic	9 (5.9)	6 (5.1)	3 (8.6)	
Arab	5 (3.3)	3 (2.5)	2 (5.7)	
HT features, *n* (%)				
LT	43 (28.1)	8 (6.8)	35 (100.0)	*p* < 0.001 ^b^
TSH	52 (34.0)	31 (26.2)	21 (60.0)	*p* < 0.001 ^b^
Anti-Tg	28 (18.3)	9 (7.6)	19 (54.2)	*p* < 0.001 ^b^
Anti-TPO	40 (26.1)	15 (12.7)	25 (71.4)	*p* < 0.001 ^b^
Positive clinical history	32 (20.9)	8 (6.8)	24 (68.6)	*p* < 0.001 ^b^
Follow-up, months, median (Q_1_, Q_3_)	58.6 (20.7, 105.4)	58.6 (18.0, 112.0)	57.1 (24.7, 99.9)	*p* = 0.67 ^a^

Abbreviations: HT, Hashimoto’s thyroiditis; Q_1_, lower quartile (25th percentile); Q_3_, upper quartile (75th percentile); LT, lymphocytic thyroiditis on surgical pathology; TSH, abnormal thyroid stimulating hormone levels; Anti-Tg, presence of anti-thyroglobulin antibodies; Anti-TPO, presence of anti-peroxidase antibodies. ^a^ Welch’s two sample *t*-test. Data analyzed on a natural log scale. ^b^ Pearson’s chi-squared test. ^c^ Fisher’s exact test.

**Table 2:** Association between Hashimoto’s thyroiditis and pediatric thyroid malignancy.

Variable	All Patients (*n* = 153)	Without HT (*n* = 118)	With HT (*n* = 35)	Statistic
Malignancy, *n* (%) ^a^	106 (69.3)	77 (65.3)	29 (82.9)	*p* = 0.05 ^b^
T, *n* (%)				*p* = 0.63 ^b^
T1 and T2	73 (68.9)	52 (67.5)	21 (72.4)	
T3 and T4	33 (31.1)	25 (32.5)	8 (27.6)	
N, *n* (%)				*p* = 0.20 ^c^
NX and N0	43 (40.6)	32 (41.6)	11 (37.9)	
N1a	18 (17.0)	10 (13.0)	8 (27.6)	
N1b	45 (42.5)	35 (45.5)	10 (34.5)	
Histopathology, *n* (%)				*p* = 0.12 *^,c^
PTC	92 (60.1)	65 (55.1)	27 (77.1)	*p* = 0.02 ^†,b^
Non-PTC	61 (39.9)	53 (44.9)	8 (22.9)	
FA	47 (30.7)	41 (34.7)	6 (17.1)	
FTC	8 (5.3)	7 (5.9)	1 (2.9)	
NIFTP	6 (3.9)	5 (4.2)	1 (2.9)	

Abbreviations: HT, Hashimoto’s thyroiditis; T, tumor stage; N, nodal stage; PTC, papillary thyroid carcinoma; FA; follicular adenoma; FTC, follicular thyroid carcinoma; NIFTP, non-invasive follicular thyroid neoplasms with papillary-like nuclear features. ^a^ Benign neoplasms included follicular adenomas; malignant neoplasms included papillary thyroid carcinoma (and variants), follicular thyroid carcinoma (and variants), and non-invasive follicular thyroid neoplasms with papillary-like nuclear features. ^b^ Pearson’s chi-squared test. ^c^ Fisher’s exact test. * test across all pathologies. ^†^ test of PTC versus non-PTC.

**Table 3:** Association between Hashimoto’s thyroiditis and pediatric papillary thyroid carcinoma subtypes.

	Without HT (*n* = 65)	With HT (*n* = 27)	Statistic
PTC subtype			*p* = 0.03 *^,a^
Classic, *n* (%)	48 (73.8)	20 (74.1)	*p* = 0.98 ^†,b^
Non-classic, (%)	17 (26.2)	7 (25.9)	
Follicular variant, *n* (%)	10 (15.4)	0 (0)	
Diffuse-sclerosing, *n* (%)	3 (4.6)	5 (18.5)	
Cribiform-morular, *n* (%)	1 (1.5)	0 (0)	
Oncocytic, *n* (%)	1 (1.5)	0 (0)	
Solid, *n* (%)	1 (1.5)	1 (3.7)	
Tall cell, *n* (%)	1 (1.5)	0 (0)	
Warthin’s-like, *n* (%)	0 (0)	1 (3.7)	

Abbreviations: HT, Hashimoto’s thyroiditis; PTC, papillary thyroid carcinoma. ^a^ Fisher’s exact test. ^b^ Pearson’s chi-squared test. * test across all pathologies. ^†^ test of classic versus non-classic papillary thyroid carcinoma.

**Table 4:** Outcomes of pediatric thyroid cancer patients with/out Hashimoto’s thyroiditis.

Variable	All Patients (*n* = 83)	Without HT (*n* = 57)	With HT (*n* = 26)	Statistic
Surgeries, *n* (%)				*p* = 0.93 ^a^
1	54 (65.1)	36 (63.2)	18 (69.2)	
2	24 (28.9)	17 (29.8)	7 (26.9)	
3	5 (6.0)	4 (7.0)	1 (3.8)	
RAI treatments, *n* (%)				*p* = 0.12 ^a^
0	29 (34.9)	19 (33.3)	10 (38.5)	
1	43 (51.8)	27 (47.4)	16 (61.5)	
2	7 (8.4)	7 (12.3)	0 (0.0)	
3	4 (4.8)	4 (7.0)	0 (0.0)	
Outcomes, *n* (%)	73 (68.9)	52 (67.5)	21 (72.4)	*p* = 0.08 ^a^
Good	58 (69.9)	36 (63.2)	22 (84.6)	
Indeterminate	7 (8.4)	7 (12.3)	0 (0.0)	
Poor	18 (21.7)	14 (24.6)	4 (15.4)	
PFS, survival rate, % (CI)				*p* = 0.99 ^b^
60 Months	86 (77–96)	86 (75–98)	86% (72–100)	
120 Months	82 (72–94)	80 (66–97)	86% (72–100)	
180 Months	82 (72–94)	80 (66–97)	86% (72–100)	
226.7 Months	82 (72–94)	NA	86% (72–100)	

Abbreviations: HT, Hashimoto’s thyroiditis; RAI, radioactive iodine; PFS, progression-free survival; CI, 95% confidence intervals; NA, not available. ^a^ Fisher’s exact test. ^b^ Log-rank test.

## Data Availability

The data that support the findings of this study are available from the corresponding author upon reasonable request.

## Supplementary Materials

The following supporting information can be downloaded at: https://www.mdpi.com/article/10.3390/cancers15194902/s1, Table S1: pathology details of patients with outcome data; Table S2: pathology details and outcomes of classic PTC patients; Figure S1: survival of pediatric classic PTC patients with/out Hashimoto’s thyroiditis.
