# Supplementary material for: Hashimoto’s Thyroiditis and the Risk of Papillary Thyroid Cancer in Children
Source: Cancers (Basel). 2023 Oct 9;15(19):4902. doi: 10.3390/cancers15194902 (PMC10572053; doi:10.3390/cancers15194902)
Supplement: Supplementary file 1 [file cancers-15-04902-s001.zip › cancers-2564068-supplementary.pdf]

Supplementary Materials for:

Hashimoto's Thyroiditis and the Risk of Papillary Thyroid Cancer in Children

Jean-Nicolas Gallant, Vivian L. Weiss, Sheau-Chiann Chen, Jiancong Liang, Ryan H. Belcher, Fei Ye, Hernan Correa, and Huiying Wang

Table S1: Pathology details of patients with outcome data 2
Table S2: Pathology details and outcomes of classic PTC patients 3
Figure S1: Survival of pediatric classic PTC patients with/out Hashimoto's thyroiditis 4

Variable	All patients (n = 83)	Without HT (n = 57)	With HT (n = 26)	Statistic
Maximal diameter, cm, median (Q_1, Q_3)	1.8 (1.0, 3.6)	2.0 (1.1, 3.5)	1.8 (1.0, 3.6)	p = 0.98 [a]
T, n (%)				p = 0.27 [b]
T1a	21 (25.3)	14 (24.6)	7 (26.9)	
T1b	19 (22.9)	10 (17.5)	9 (34.6)	
T2	15 (18.1)	13 (22.8)	2 (7.7)	
T3a	15 (18.1)	9 (15.8)	6 (23.1)	
T3b	7 (8.4)	6 (10.5)	1 (3.8)	
T4a	6 (7.2)	5 (8.8)	1 (3.8)	
N, n (%)				p = 0.20 [b]
NX	12 (14.5)	10 (17.5)	2 (7.7)	
N0	16 (19.3)	9 (15.8)	7 (26.9)	
N1a	15 (18.1)	8 (14.0)	7 (26.9)	
N1b	40 (48.2)	30 (52.6)	10 (38.5)	
M, n (%)	3 (3.6)	3 (5.3)	0 (0.0)	p = 0.55 [b]
Multifocal disease, n (%)	37 (44.6)	25 (43.9)	12 (46.2)	p = 0.85 [c]
Extra-thyroidal extension, n (%)				p = 0.77 [b]
Microscopic	12 (14.5)	9 (15.8)	3 (11.5)	
Gross	2 (2.4)	2 (3.5)	0 (0.0)	
Extra-nodal extension, n (%)	23 (62.2)	18 (66.7)	5 (50.0)	p = 0.45 [b]
Vascular invasion, n (%)				p = 0.40 [c]
Present	17 (21.2)	13 (24.1)	4 (15.4)	
Extensive	18 (22.5)	10 (18.5)	8 (30.8)	
Lymphatic invasion, n (%)	36 (44.4)	24 (43.6)	12 (46.2)	p = 0.83 [c]
ATA, n (%)				p = 0.40 [b]
Low	39 (47.0)	24 (42.1)	15 (57.7)	
Intermediate	8 (9.6)	6 (10.5)	2 (7.7)	
High	36 (43.4)	27 (47.4)	9 (34.6)	

Table S1: Pathology details of patients with outcome data
Abbreviations: HT, Hashimoto's thyroiditis; Q_1, lower quartile (25th percentile); Q_3, upper quartile (75th percentile); T, tumor stage; N, nodal stage; M, presence of distant metastases; ATA, American Thyroid Association risk category

[a] Welch's two sample t-test. Data are analyzed on a natural log scale.
[b] Fisher's exact test
[c] Pearson's Chi-squared test

Variable	Classic PTC (n = 59)	Without HT (n = 41)	With HT (n = 18)	Statistic
Maximal diameter, cm, median (Q_1, Q_3)	1.6 (0.8, 3.0)	1.6 (1.0, 3.2)	1.6 (0.7, 1.9)	p = 0.64 [a]
T, n (%)				p = 0.13 [b]
T1a	18 (30.5)	12 (29.3)	6 (33.3)	
T1b	16 (27.1)	8 (19.5)	8 (44.4)	
T2	8 (13.6)	8 (19.5)	0 (0.0)	
T3a	9 (15.3)	6 (14.6)	3 (16.7)	
T3b	4 (6.8)	4 (9.8)	0 (0.0)	
T4a	4 (6.8)	3 (7.3)	1 (5.6)	
N, n (%)				p = 0.02 [b]
NX	10 (16.9)	8 (19.5)	2 (11.1)	
N0	10 (16.9)	4 (9.8)	6 (33.3)	
N1a	15 (25.4)	8 (19.5)	7 (38.9)	
N1b	24 (40.7)	21 (51.2)	3 (16.7)	
M, n (%)	2 (3.4)	2 (11.1)	0 (0.0)	p = 0.99 [c]
Multifocal disease, n (%)	26 (44.0)	18 (43.9)	8 (44.4)	p = 0.97 [c]
Extra-thyroidal extension, n (%)				p = 0.57 [b]
Microscopic	7 (11.9)	6 (14.6)	1 (5.6)	
Gross	2 (3.4)	2 (11.1)	0 (0.0)	
Extra-nodal extension, n (%)	16 (27.1)	13 (31.7)	3 (16.7)	p = 0.48 [c]
Vascular invasion, n (%)				p = 0.85 [b]
Present	13 (22.0)	10 (24.4)	3 (16.7)	
Extensive	9 (15.3)	6 (14.6)	3 (16.7)	
Lymphatic invasion, n (%)	22 (37.2)	16 (39.0)	6 (33.3)	p = 0.63 [c]
ATA, n (%)				p = 0.04 [b]
Low	31 (52.5)	17 (41.4)	14 (77.8)	
Intermediate	6 (10.2)	5 (12.2)	1 (5.6)	
High	22 (37.3)	19 (46.3)	3 (16.7)	
Surgeries, n (%)	1 (1–2)	1 (1–2)	1 (1–2)	p = 0.46 [b]
1	41 (69.5)	28 (68.3)	13 (72.2)	
2	14 (23.7)	9 (22.0)	5 (27.8)	
3	4 (6.8)	4 (9.8)	0 (0.0)	
RAI treatments, n (%)				p = 0.33 [a]
0	22 (37.3)	13 (31.7)	9 (50.0)	
1	30 (50.8)	21 (51.2)	9 (50.0)	
2	4 (6.8)	4 (9.8)	0 (0.0)	
3	3 (5.1)	3 (7.3)	0 (0.0)	
Outcomes, n (%)				p = 0.03 [b]
Good	42 (71.2)	25 (61.0)	17 (94.4)	
Indeterminate	5 (8.5)	5 (12.2)	0 (0.0)	
Poor	12 (20.3)	11 (26.8)	1 (5.6)	
PFS, survival rate, % (CI)				p = 0.68 [d]
60 Months	88 (77–100)	85 (71–100)	93 (80–100)	
120 Months	88 (77–100)	85 (71–100)	93 (80–100)	
180 Months	88 (77–100)	85 (71 - 100)	93 (80–100)	
226.7 Months	88 (77–100)	NA	93 (80–100)	

Table S2: Pathology details and outcomes of classic PTC patients
Abbreviations: HT, Hashimoto's thyroiditis; Q_1, lower quartile (25th percentile); Q_3, upper quartile (75th percentile); T, tumor stage; N, nodal stage; M, presence of distant metastases; ATA, American Thyroid Association risk category; RAI, radioactive iodine; PFS, progression-free survival; CI, 95% confidence intervals; NA, not available.

[a] Welch's two sample t-test. Data are analyzed on a natural log scale.
[b] Fisher's exact test
[c] Pearson's Chi-squared test
[d] Log-rank test

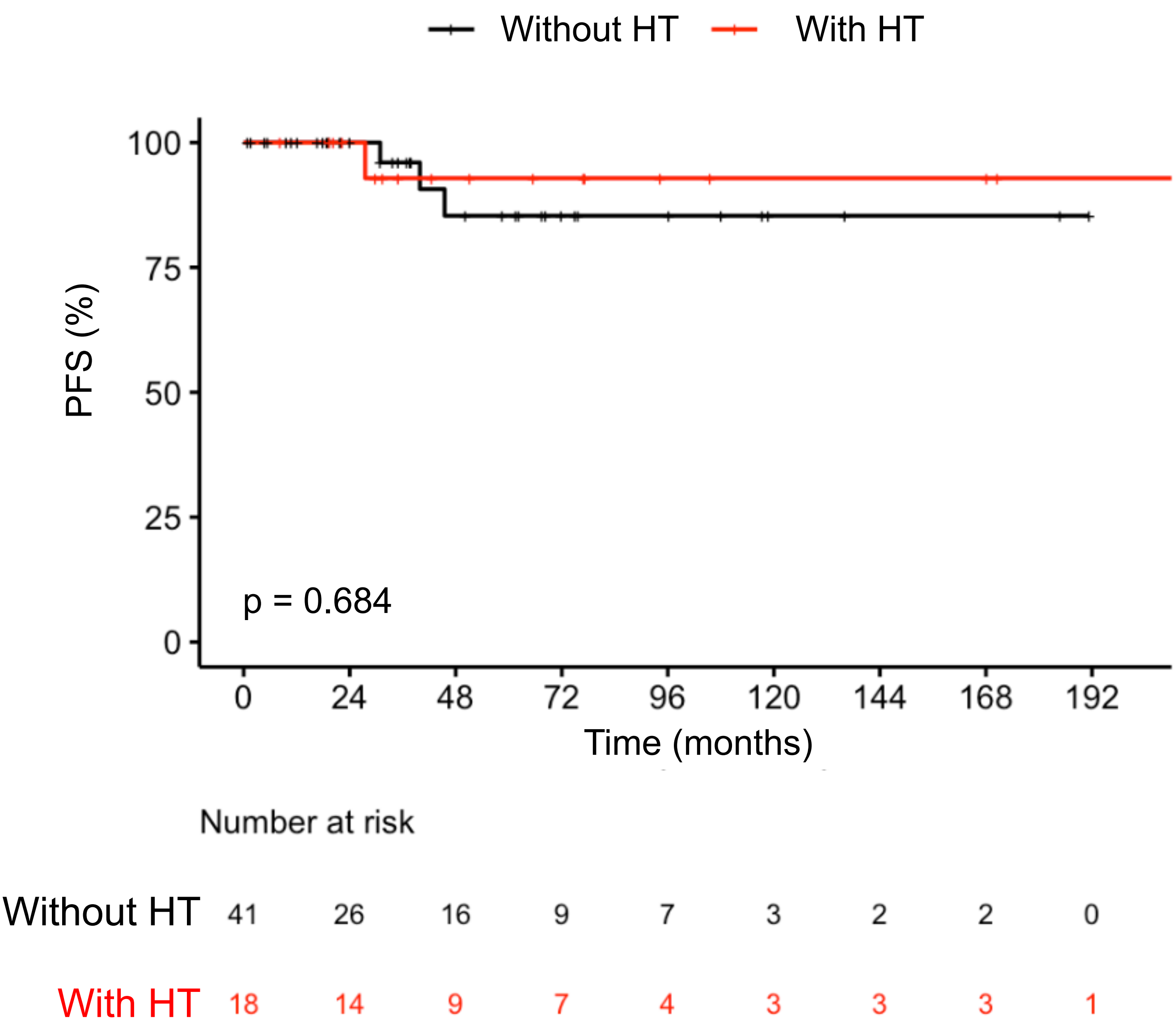

Figure S1: Survival of pediatric classic PTC patients with/out Hashimoto's thyroiditis
Abbreviations: HT, Hashimotos' thyroiditis; PFS, progression-free survival; ; p, p-value from log-rank test used to compare the PFS of groups.
